# Fasting-induced RNF152 resensitizes gallbladder cancer cells to gemcitabine by inhibiting mTORC1-mediated glycolysis

**DOI:** 10.1016/j.isci.2024.109659

**Published:** 2024-04-08

**Authors:** Ying Tao, Zijun Gong, Sheng Shen, Yaqi Ding, Rui Zan, Bohao Zheng, Wentao Sun, Chaolin Ma, Mengxuan Shu, Xiao Lu, Han Liu, Xiaoling Ni, Houbao Liu, Tao Suo

**Affiliations:** 1 Department of General Surgery, Shanghai Xuhui Central Hospital, Zhongshan-Xuhui Hospital, Fudan University, Shanghai, China; 2 Department of Biliary Surgery, Zhongshan Hospital, Fudan University, Shanghai, China; 3 Shanghai Engineering Research Center of Biliary Tract Minimal Invasive Surgery and Materials, Shanghai, China; 4 Biliary Tract Disease Institute, Fudan University, Shanghai, China; 5 The Center of Biliary Disease Center, Zhongshan Hospital, Fudan University, Shanghai, China; 6 Ruijin Hospital LuWan Branch, Shanghai Jiao Tong University School of Medicine Central Laboratory, Shanghai, China

**Keywords:** Molecular biology, Cell biology, Cancer

## Abstract

Abnormal mTORC1 activation by the lysosomal Ragulator complex has been implicated in cancer and glycolytic metabolism associated with drug resistance. Fasting upregulates RNF152 and mediates the metabolic status of cells. We report that RNF152 regulates mTORC1 signaling by targeting a Ragulator subunit, p18, and attenuates gemcitabine resistance in gallbladder cancer (GBC). We detected levels of RNF152 and p18 in tissues and undertook mechanistic studies using activators, inhibitors, and lentivirus transfections. RNF152 levels were significantly lower in GBC than in adjacent non-cancer tissues. Fasting impairs glycolysis, induces gemcitabine sensitivity, and upregulates RNF152 expression. RNF152 overexpression increases the sensitivity of GBC cells to gemcitabine, whereas silencing RNF152 has the opposite effect. Fasting-induced RNF152 ubiquitinates p18, resulting in proteasomal degradation. RNF152 deficiency increases the lysosomal localization of p18 and increases mTORC1 activity, to promote glycolysis and decrease gemcitabine sensitivity. RNF152 suppresses mTORC1 activity to inhibit glycolysis and enhance gemcitabine sensitivity in GBC.

## Highlights

• Abnormal mTORC1 activation linked to cancer and drug resistance metabolism
• Fasting boosts RNF152, regulating cell metabolism
• RNF152 targets Ragulator subunit, attenuating gemcitabine resistance
• RNF152 ubiquitinates p18, inhibiting mTORC1 and enhancing drug sensitivity

## Graphical abstract

**Figure:**
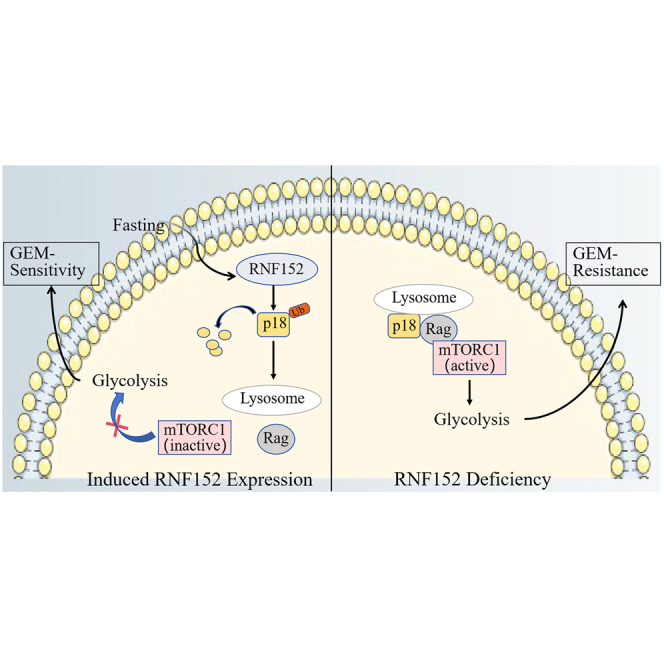

## Introduction

Gallbladder cancer (GBC), despite being one of the most common malignancies of the biliary tract, is often diagnosed at advanced stages when the prognosis is poor.^1^^,^^2^ The early detection of GBC is limited because it is mostly asymptomatic and is often diagnosed when metastatic dissemination has occurred.^3^ These challenges are further compounded by limited treatment options because GBC develops resistance to most chemotherapies.^4^^,^^5^

Increasing evidence suggests that atypical physiological conditions such as hyperglycemia and a heterogeneous tumor microenvironment contribute to drug resistance by creating the metabolic conditions that support the proliferation of cancer cells.^6^^,^^7^^,^^8^ In healthy tissues, cells obtain energy through mitochondrial oxidative phosphorylation, whereas the increased proliferation associated with tumor cells is derived from energy obtained by aerobic glycolysis.^9^ This is known as the Warburg effect and a well-known characteristic of cancerous cells,^10^^,^^11^ including GBC.^12^ For instance, hyperglycemia before surgery in GB C is known as a risk factor for overall survival (OS).^13^ Therefore, controlling hyperglycemia or targeting metabolic pathways in cancer cells to enhance sensitivity to chemotherapeutic agents has become an important strategy in cancer management.^14^

Fasting is known to modulate the upregulation of metabolic pathways in cancer cells.^15^ Intermittent fasting by consuming no or minimal amounts of food for a specific duration of time, usually 12 to 24 h, can decrease the growth of primary tumors in animal models.^16^^,^^17^ Fasting has also been found to reduce proliferation and glucose uptake in the tumor cells of GBC and other biliary tract cancers.^18^^,^^19^ The reduction of glucose in cells through fasting is believed to inhibit AKT/mTOR signaling to prevent the Warburg shift and enhance cancer cell sensitivity to chemotherapy.^18^^,^^20^ In addition, during nutrient deprivation, healthy cells cease proliferating and divert their resources to maintenance, whereas tumor cells continue to divide because of mutations in tumor suppressor genes and mitogenic pathways. This distinct response to fasting between healthy and tumor cells is known as differential stress resistance and contributes to tumor susceptibility to chemotherapeutic agents.^21^^,^^22^

RING finger-related E3 ubiquitin ligases target specific proteins for degradation by mediating the covalent attachment of ubiquitin.^23^ They contribute to tumorigenesis, cancer development, and drug resistance and display both oncogenic and tumor-suppressive functions.^24^ The RING finger protein 152 (RNF152) was detected in an unbiased genome-wide CRISPR-Cas9 loss of function screen to identify genes involved in gemcitabine (GEM) resistance in GBC.^25^ RNF152 is known to be downregulated in several biliary tract cancers, including hepatocellular carcinoma and colorectal cancer.^26^^,^^27^ In starved cells, RNF152-related protein degradation leads to the inactivation of the mTOR complex 1 (mTORC1), which is involved in the multidrug resistance of GBC.^28^^,^^29^ Increased levels of mTORC1 are known to enrich levels of the Ragulator complex protein p18/LAMTOR1, which anchors mTORC1 to lysosomal membranes.^30^^,^^31^ Once attached to lysosomes, mTORC1 plays a pivotal role in cell metabolism and activating the translation of proteins involved in glycolysis and drug resistance.

**Scheme 1:**
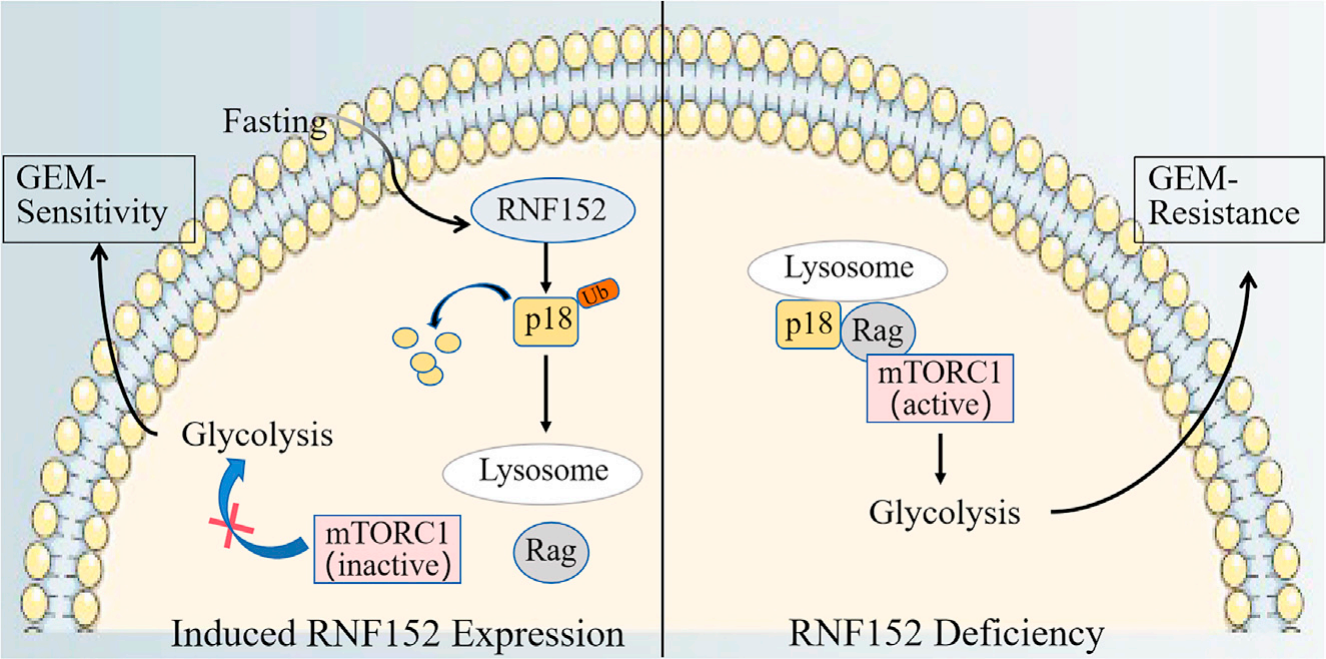
This model suggests that during fasting, RNF152 is upregulated, promoting the ubiquitination and degradation of p18 while inhibiting its interaction with Rag. Consequently, this disruption prevents the recruitment of mTORC1 for activation on the lysosome. In RNF152-deficient cells, the formation of the p18-Rag complex on lysosomes occurs, resulting in the activation of mTORC1 and gemcitabine resistance.

In this study, we determined the relationship between p18, RNF152, and mTORC1 in GBC. RNF152 expression levels were determined in the tissue of patients with GBC and GBC cells grown in fasting mimic media.^18^ We found a lower level of RNF152 expression in GBC cells and tissue. However, fasting upregulates the expression of RNF152, which then targets p18/LAMTOR1 to inhibit the activity of mTORC1 and thereby increase the sensitivity of GBC cells to GEM. Our results indicate that fasting or the overexpression of RNF152 could be used with GEM to manage GBC more effectively, as shown in Scheme 1.

## Results

### Fasting inhibits glycolysis and promotes GEM sensitivity in GBC cells

**Figure 1:**
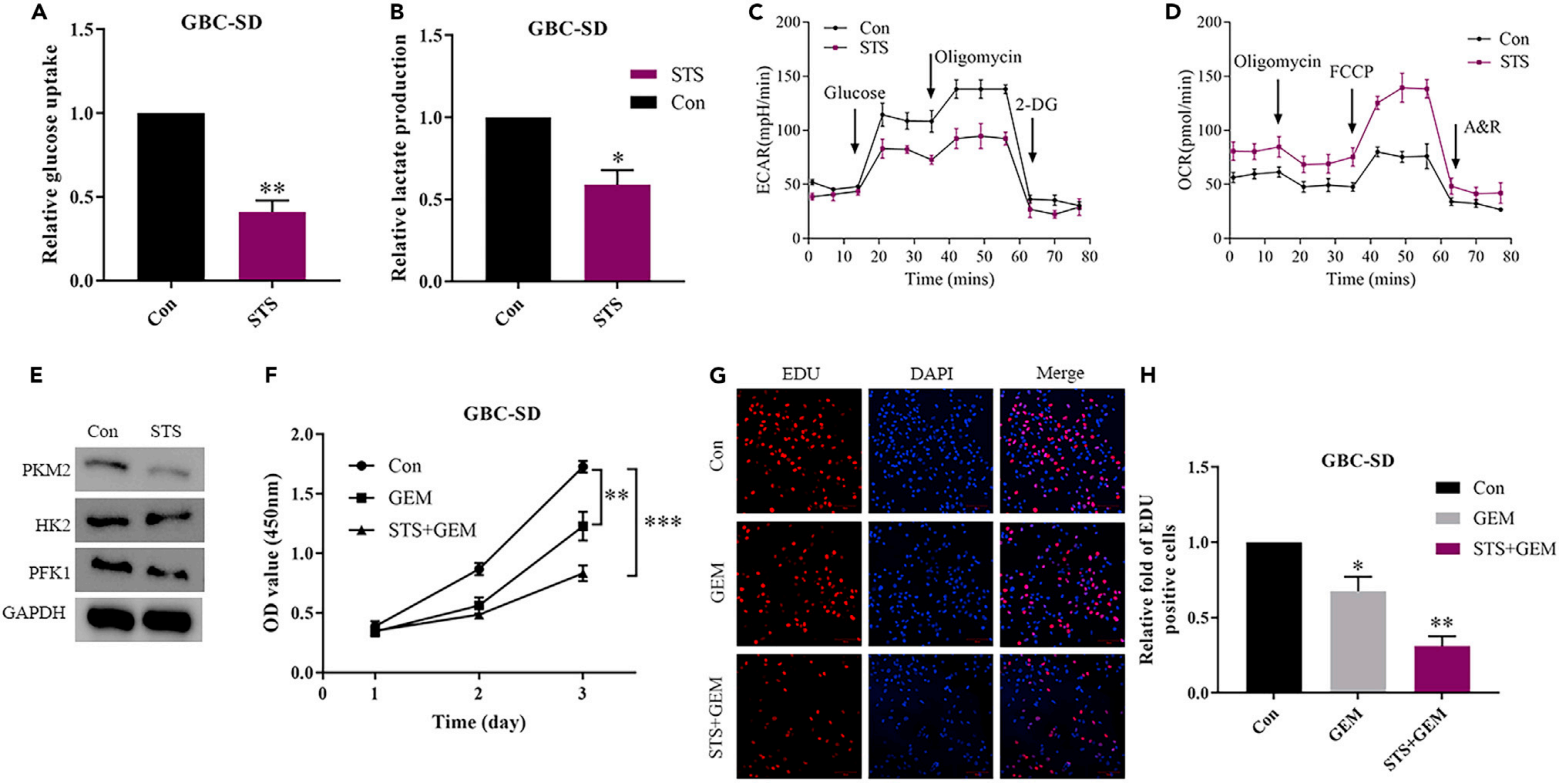
Fasting impairs glycolysis and induces gemcitabine sensitivity in gallbladder cancer (GBC) (A) Short-term starvation (STS) reduced glucose uptake in GBC-SD cells. (B) STS decreased lactate production via glycolysis in GBC-SD cells. (C) Extracellular acidification rate (ECAR), an indicator of glycolysis, was reduced in GBC-SD cells cultured in fasting mimic medium. (D) Oxygen consumption rate (OCR), which reflects mitochondrial respiration, was increased in GBC-SD cells cultured in fasting mimic medium. (E) STS downregulates the expression of rate-limiting glycolytic enzymes in glucose metabolism (PKM2, HK2, and PFK1) according to western blot analysis. (F) STS sensitizes cancer cells to gemcitabine (GEM)-mediated cytotoxic effect as measured by a CCK8 assay. (G) Cell proliferation was also evaluated using EdU immunofluorescence staining. (H) The graph shows the relative fold fraction of EdU-positive cells. Data presented as Means ± SD (*n* = 3). ∗*p* < 0.05, ∗∗*p* < 0.01 compared with the control group.

To verify that fasting can influence the metabolic status and drug resistance of cancer cells, we measured glucose uptake and lactate production in GBC-SD cells cultured in either fasting mimic media (i.e., short-term starvation, STS) or regular media (control) for 48 h (Figures 1A and 1B). STS significantly decreased glucose uptake and lactate production in GBC-SD cells. Extracellular acidification rate (ECAR) measurements indicated that glycolysis was reduced in GBC-SD cells cultured in the fasting mimic medium (Figure 1C) and oxygen consumption rate (OCR), which measures the extent of mitochondrial respiration, was increased (Figure 1D). The expression of rate-limiting glycolytic enzymes (PKM2, HK2, and PFK1) in the GBC-SD cells substantiate these results (Figure 1E). STS downregulates the expression of PKM2, HK2, and PFK1 and signifies a reduction in glucose metabolism. Furthermore, STS also sensitizes cancer cells to the cytotoxic effects of GEM (Figures 1F–1H). Measuring cell numbers and proliferation by CCK8 assay and EdU immunofluorescence staining demonstrates that GEM-treated STS GBC-SD cells have lower cell viability than control cells. Our results reinforce the evidence that fasting reduces levels of glycolysis and renders GBC cells more susceptible to chemotherapeutic agents. For instance, Krstic et al. reported that fasting has a sorafenib-sensitizing effect on hepatocellular carcinoma through p53-dependent metabolic synergism.^20^ Similarly, fasting can enhance the susceptibility of breast cancer cells to doxorubicin and cyclophosphamide and can protect normal cells from chemotoxicity by reducing levels of glycolysis.^22^

### RNF152 expression is suppressed in GBC

**Figure 2:**
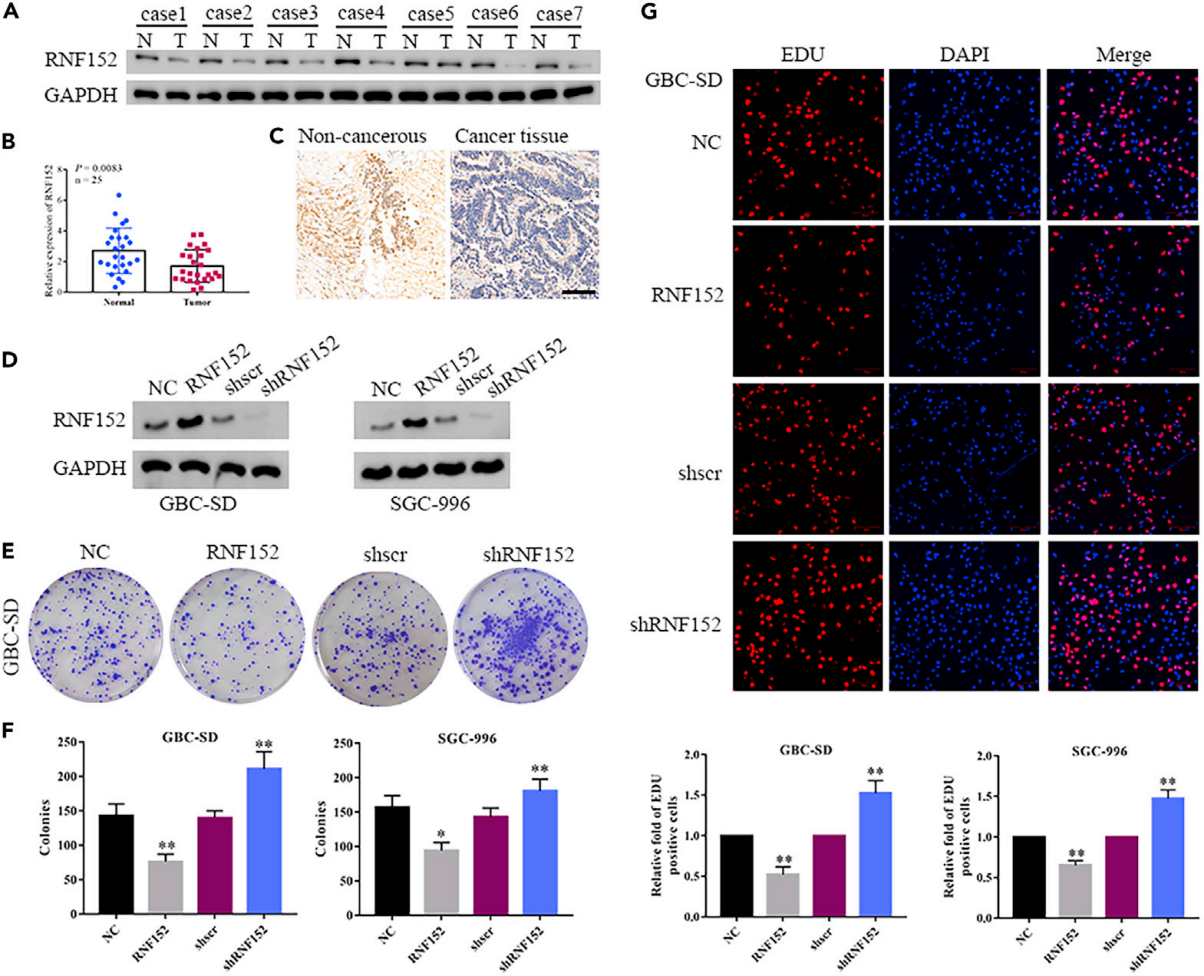
RNF152 is a potential therapeutic target for the treatment of gallbladder cancer (GBC) (A) Protein expression levels of RNF152 in seven patients with GBC were measured by western blot analysis of the paired GBC and peritumoral tissue samples. (B) Relative mRNA expression of RNF152 in 25 paired GBC tissues, as determined by RT-PCR. (C) Representative immunohistochemical (IHC) images showing RNF152 expression in GBC tissues and non-tumor tissue from patients with GBC. Scale bar, 100 μm. (D) Western blot analysis of the RNF152 protein levels in GBC-SD and SGC-996 cells after transfection. NC, negative control. (E) Colony formation assay of cell proliferation in GBC-SD lines after transfection. (F) The graph shows the statistical results of the colonies in GBC-SD and SGC-996 cells. (G) Cell proliferation was also evaluated in the indicated cells using EdU staining. Scale bar: 100 μm. Data presented as Means ± SD (*n* = 3). ∗∗*p* < 0.01 compared with the NC or shScr group.

To determine the status of RNF152 in GBC we measured levels of expression in the tumor tissues of 25 patients with GBC and examined its correlation with clinical parameters (Table 1). The protein levels of RNF152 and mRNA expression were significantly lower in GBC tumor tissue than in adjacent non-cancerous tissue (*p* = 0.0083) (Figures 2A and 2B). Staining by immunohistochemistry (IHC) detected higher levels of RNF152 in normal tissues than in tumor tissue (Figure 2C). To further investigate the role played by RNF152 in GBC, we altered its expression in GBC-SD and SGC-996 cells (Figure 2D). Colony formation assays indicated that cell proliferation was significantly reduced when RNF152 is overexpressed in GBC cells, whereas silencing RNF152 resulted in enhanced levels of cell proliferation (Figures 2E and 2F). These results were confirmed by EdU staining (Figure 2G). Overall, these findings demonstrate that inhibiting the expression of RNF152 enhances the proliferation of GBC cells.

**Table 1:** Correlation of RNF152 expression and clinic parameters in 25 patients of gallbladder cancer

Characteristics	All cases	RNF152		*p* value
		Low	High	
Participants	25	9	16	
Age (years)				0.678
<60	11	4	7	
≥60	14	4	10	
Gender				0.610
Male	10	3	7	
Female	15	6	9	
Clinical stage				0.174
1–2	9	2	7	
3–4	16	8	8	
Differentiation				0.033
Well	10	7	3	
Poorly	15	4	11	
Lymphnode metastasis				0.008
No	7	5	2	
Yes	18	3	15

### Downstream regulation of RNF152 is influenced by fasting in GBC

**Figure 3:**
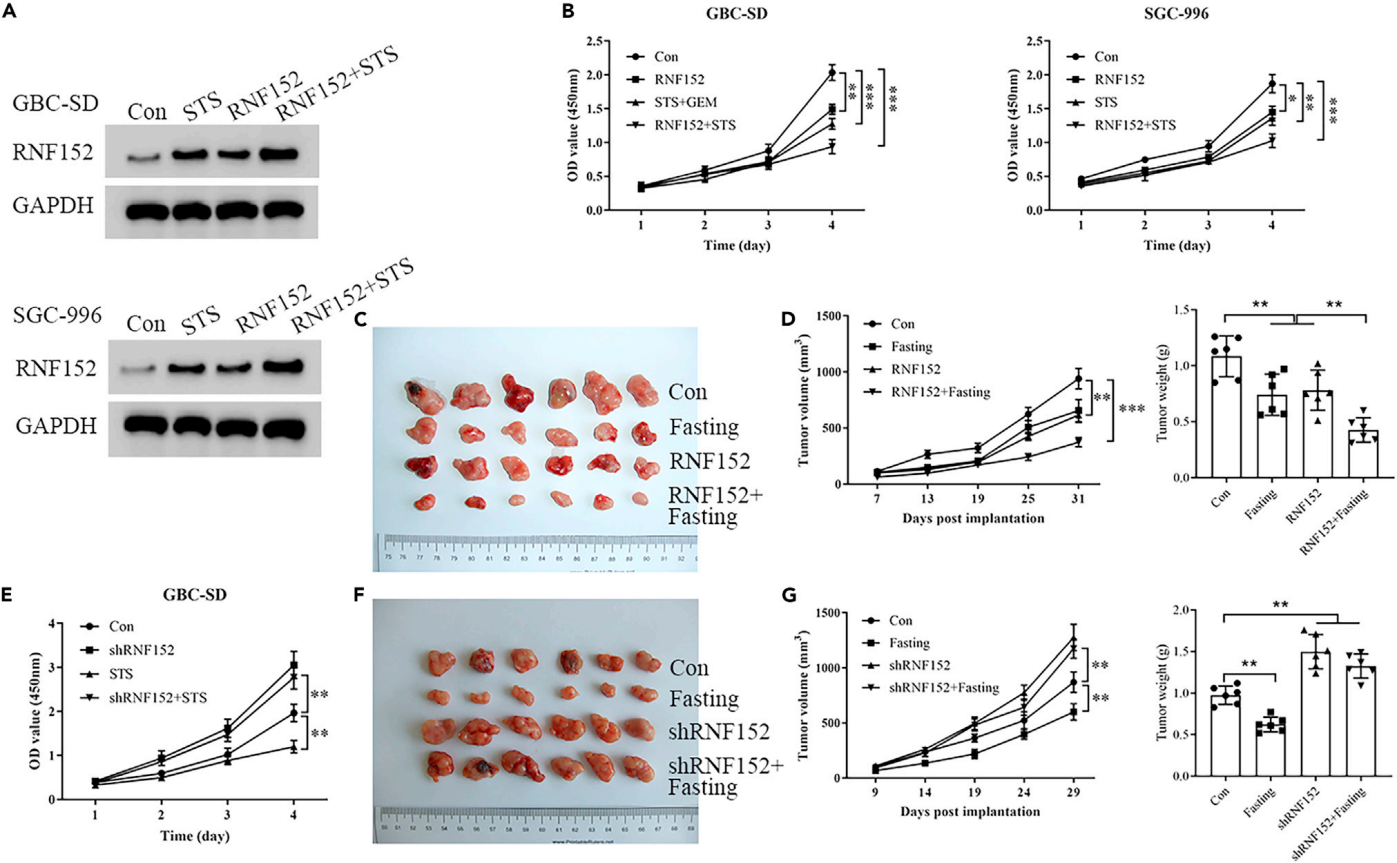
RNF152 is an important downstream target of fasting in gallbladder cancer (GBC) (A) Western blotting indicated that STS for 48 h and RNF152 overexpression increased the protein level of RNF152. (B) RNF152 overexpression and STS for 48 h inhibited GBC cell proliferation as measured by a CCK8 assay. (C) Images of dissected tumors. Both fasting and RNF152 overexpression inhibited tumor growth in the mice. Fasting combined with the implantation of RNF152-overexpressing GBC-SD cells had the most obvious inhibitory effect on tumor growth in the mice, *n* = 6 per group. (D) Tumor volumes and tumor weights. (E) The effect of RNF152 knockdown, STS 48 h, and RNF152 knockdown combined with STS on GBC-SD cell proliferation was evaluated by CCK8. (F) Images of dissected tumors. Fasting inhibited tumor growth in mice. RNF152 knockdown promoted tumor growth in mice. Fasting combined with shRNF152 GBC-SD cells did not inhibit tumor growth in the mice, *n* = 6 per group. (G) Tumor volumes and tumor weights. ∗∗*p* < 0.01, ∗∗∗*p* < 0.001.

To determine if fasting can influence the downstream regulation of RNF152 in GBC, we measured its expression in cells cultured in fasting mimic media for 48 h. Western blotting indicated that STS for 48 h increased the protein levels of RNF152 and reduced levels of cell proliferation (Figures 3A and 3B). When these cancer cells were transplanted in mice to create a xenograft model of GBC, fasting and the overexpression of RNF152 resulted in tumors with a smaller mass (Figures 3C and 3D). Both fasting and RNF152 overexpression inhibited tumor growth. Fasting combined with the implantation of RNF152-overexpressing GBC-SD cells had the most obvious inhibitory effect on tumor growth in the mice (Figures 3C and 3D). In contrast, suppressing the expression of RNF152 partially reverses the effects of fasting (Figures 3E–3G). Therefore, fasting promotes the expression of RNF152 and inhibits the growth of GBC *in vitro* and *in vivo*, indicating that fasting could influence the downstream regulation of RNF152.

### Loss of RNF152 decreases GEM sensitivity

**Figure 4:**
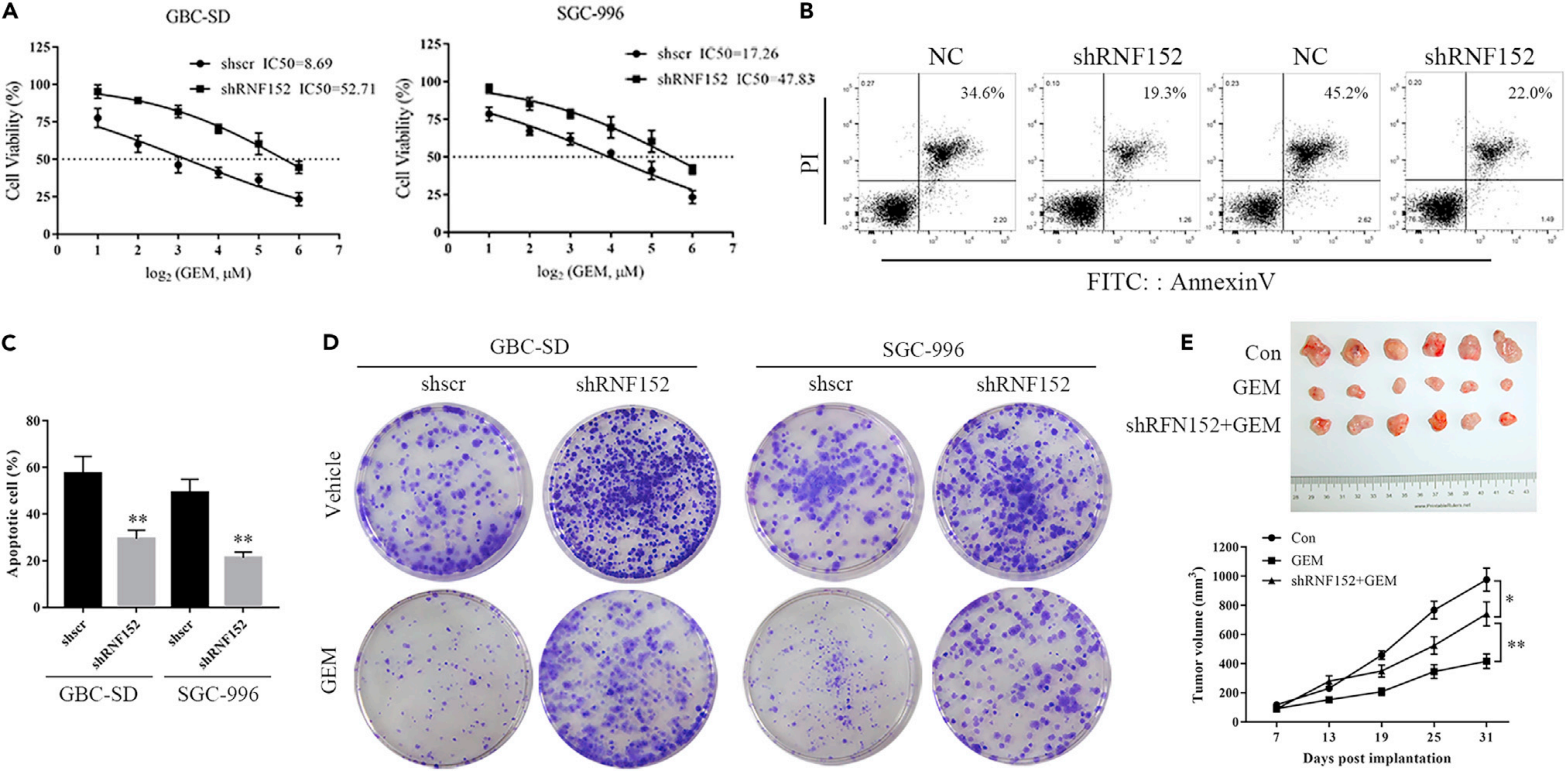
Loss of RNF152 decreases gemcitabine (GEM) sensitivity *in vitro* and *in vivo* (A and B) RNF152-depleted cells exhibited resistance to GEM, as assessed by cell viability assay under GEM treatment for 72 h at indicated doses (A) and apoptotic assay by Annexin-V/PI staining for flow cytometry after GEM treatment for 72 h at IC50 (B). (C) The graph shows the statistical results of apoptosis. (D) Representative images of cell densities in GBC-SD and SGC-996 cells treated with GEM at IC50 or vehicle and stained with crystal violet. (E) Images and volume of tumors isolated from mice in different groups. ∗*p* < 0.05, ∗∗*p* < 0.01.

GBC cells are known to acquire increased resistance to chemotherapy. To verify whether the expression of RNF152 could influence drug sensitivity in GBC, we determined the IC50 in GBC cells treated with GEM and lacking RNF152 expression. The level of GEM resistance was significantly increased in GBC-SD (control IC50 = 8.69, shRNF152 IC50 = 52.71) and SGC996 (control IC50 = 17.26, shRNF152 IC50 = 47.83) cells lacking the expression of RNF152 (Figure 4A). Apoptotic assays confirmed these results (Figures 4B and 4C). Higher levels of apoptotic cells were observed in the control cells transfected with shscr (GB-SD = 34.6%, SGC996 = 45.2%) than in those transfected with shRNF152 (GB-SD = 19.3%, SGC996 = 22.09%). Representative images of cell densities in GBC-SD and SGC-996 cells treated with an IC50 concentration of GEM illustrate that the depletion of RNF152 results in increased levels of drug resistance (Figure 4D). RNF152 deletion also reduces the sensitivity to GEM *in vivo* (Figure 4E). Tumors expressing RNF152 are smaller and have an increased response to GEM than those inhibiting the expression of RNF152.

### RNF152 represses mTORC1-mediated glycolysis during fasting

**Figure 5:**
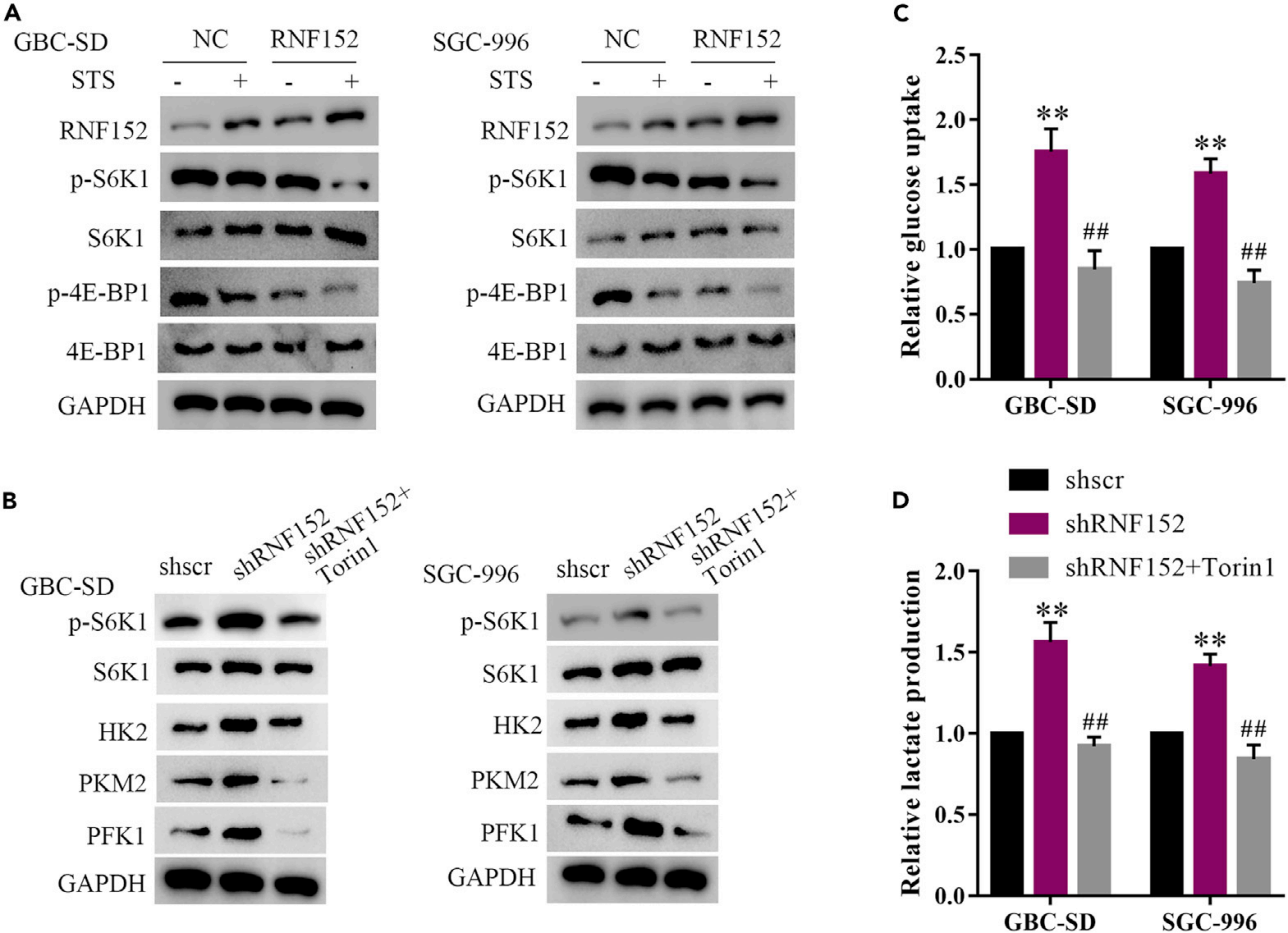
RNF152 senses fasting to repress mTORC1-mediated glycolysis (A) Both STS and RNF152 overexpression inhibited mTORC1 activation (thereby downregulating p-4E-BP1 and pS6K1). STS combined with RNF152 overexpression had the most obvious inhibitory effect on mTORC1 activation. (B) RNF152 knockdown promoted glycolysis and increased mTORC1 activation, and this effect could be reversed by the mTOR inhibitor Torin1 (250 nM). (C) RNF152 knockdown increased glucose uptake, and this increase could be reversed by Torin1. (D) RNF152 knockdown increased lactate production via glycolysis, and this increase could also be reversed by Torin1. ∗∗*p* < 0.01 compared with the shScr group, ##*p* < 0.01 compared with the shRNF152 group.

Glycolysis is believed to be mediated in cancer cells through the mTORC1 pathway and involves the activity of p-4E-BP1 and pS6K1.^31^^,^^32^ Therefore, we determined the levels of these proteins in GBC cells grown under STS with or without the overexpression of RNF152. The downregulation of p-4E-BP1 and pS6K1 indicated that both STS and RNF152 overexpression inhibited the mTORC1 pathway and STS combined with RNF152 overexpression had the most obvious inhibitory effect on mTORC1 activation (Figure 5A). In contrast, RNF152 knockdown promoted glycolysis and increased mTORC1 activation but this effect could be reversed by the mTOR inhibitor Torin1 (Figure 5B). RNF152 knockdown increased glucose uptake and lactate production but this increase in glycolysis could also be reversed by Torin1 (Figures 5C and 5D). All these factors combined indicate that increased levels of RNF152 through STS are associated with the suppression of mTORC1-mediated glycolysis.

### RNF152 regulates p18 levels in a proteasome-dependent manner

**Figure 6:**
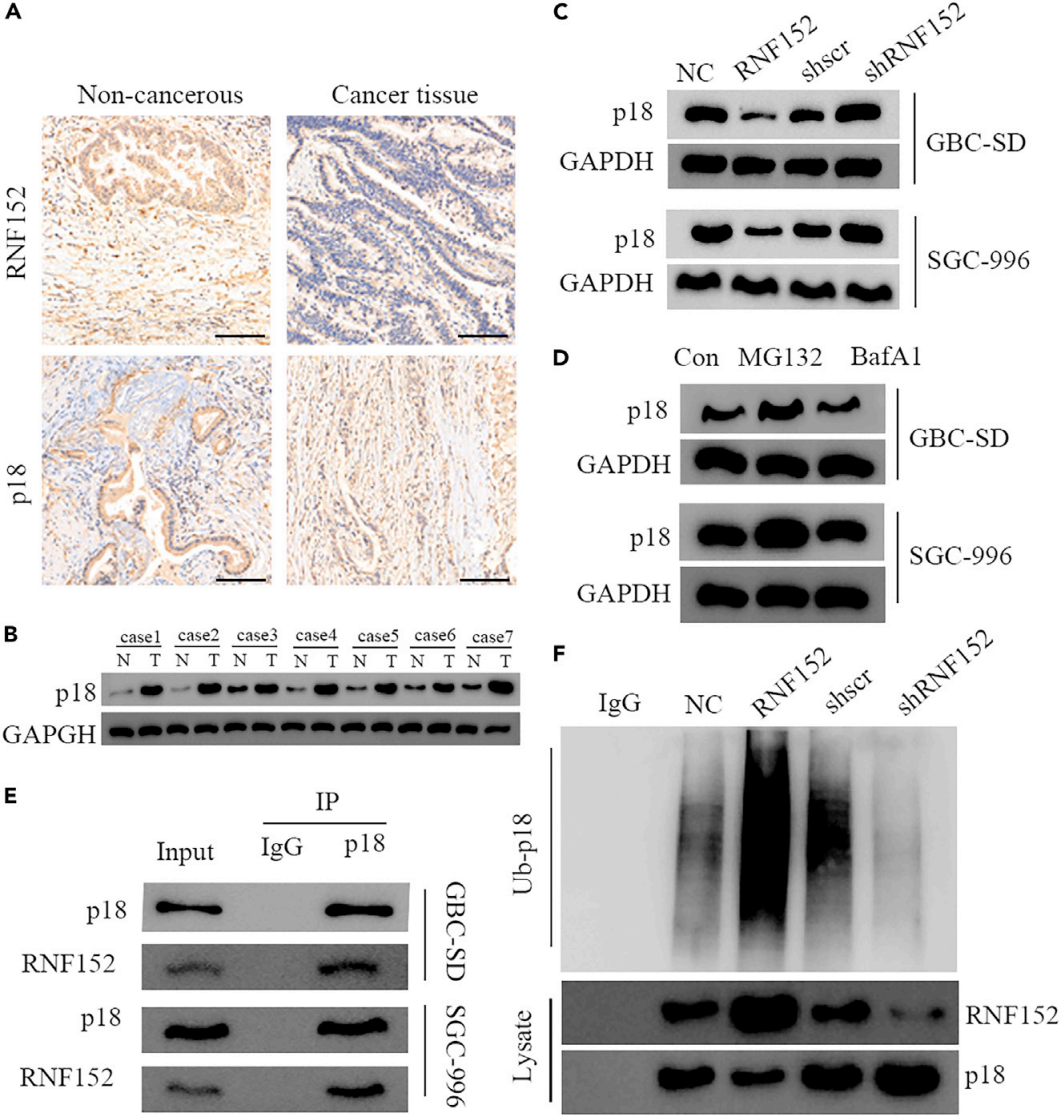
RNF152 regulates p18 levels in a proteasome-dependent manner (A) Representative immunohistochemical (IHC) images illustrating levels of RNF152 and p18 expression in GBC tissues and non-tumor tissue. Scale bar, 100 μm. (B) Western blot analysis of p18 protein expression levels in GBC and matched normal tissues. (C) p18 expression following RNF152 knockdown and overexpression was quantified by western blotting. (D) A proteasome inhibitor, MG132 (10 mM), or a lysosome inhibitor, the vacuolar H + -ATPase inhibitor, bafilomycin A1 (BafA, 100 nM), for 30 min. MG132, but not BafA, significantly increased p18 levels. (E) Immunoprecipitation analysis with the indicated antibodies (exogenous interaction). The endogenous interaction of RNF152 and p18 was identified in GBC-SD and SGC-996 cells. (F) Representative western blot images of p18 immunoprecipitated and analyzed by anti-ubiquitin antibody.

Increased levels of mTORC1 are associated with enriched levels of the Ragulator complex protein p18, which anchors mTORC1 to lysosomal membranes.^31^ Therefore, we investigated whether the differential expression of RNF152 may affect levels of p18. We found an opposing expression profile between RNF152 and p18 in GBC tissue. Representative IHC images indicate low levels of RNF152 and high levels of p18 expression in GBC tissues, whereas high levels of RNF152 and low levels of p18 expression are found in non-tumor tissues (Figure 6A). Western blot analysis of p18 protein expression levels in GBC and matched normal tissues confirm these results (Figure 6B). Moreover, when RNF152 is inhibited, levels of p18 increase, whereas the overexpression of RNF152 lowers the expression of p18 (Figure 6C). Western blotting analysis of p18 after the addition of a proteasome inhibitor, MG132 (10 mM), or a lysosome inhibitor, bafilomycin A1 (BafA, 100 nM, a V-ATPase inhibitor), indicated that the levels of p18 are increased by MG132. This suggests that levels of p18 are regulated by the proteasome (Figure 6D). Immunoprecipitation (IP) analysis suggests that an endogenous interaction exists between RNF152 and p18 in GBC-SD and SGC-996 cells (Figure 6E). Western blot analysis of p18 immunoprecipitated from cells over or under-expressing RNF152 and analyzed by anti-ubiquitin antibody indicates a greater degree of ubiquitination when RNF152 levels are higher (Figure 6F). Overall, these results indicate that RNF152 regulates the levels of p18 in a proteasome-dependent manner.

### Increased p18 levels are associated with the increased lysosomal localization of the Ragulator-Rag complex and mTORC1

**Figure 7:**
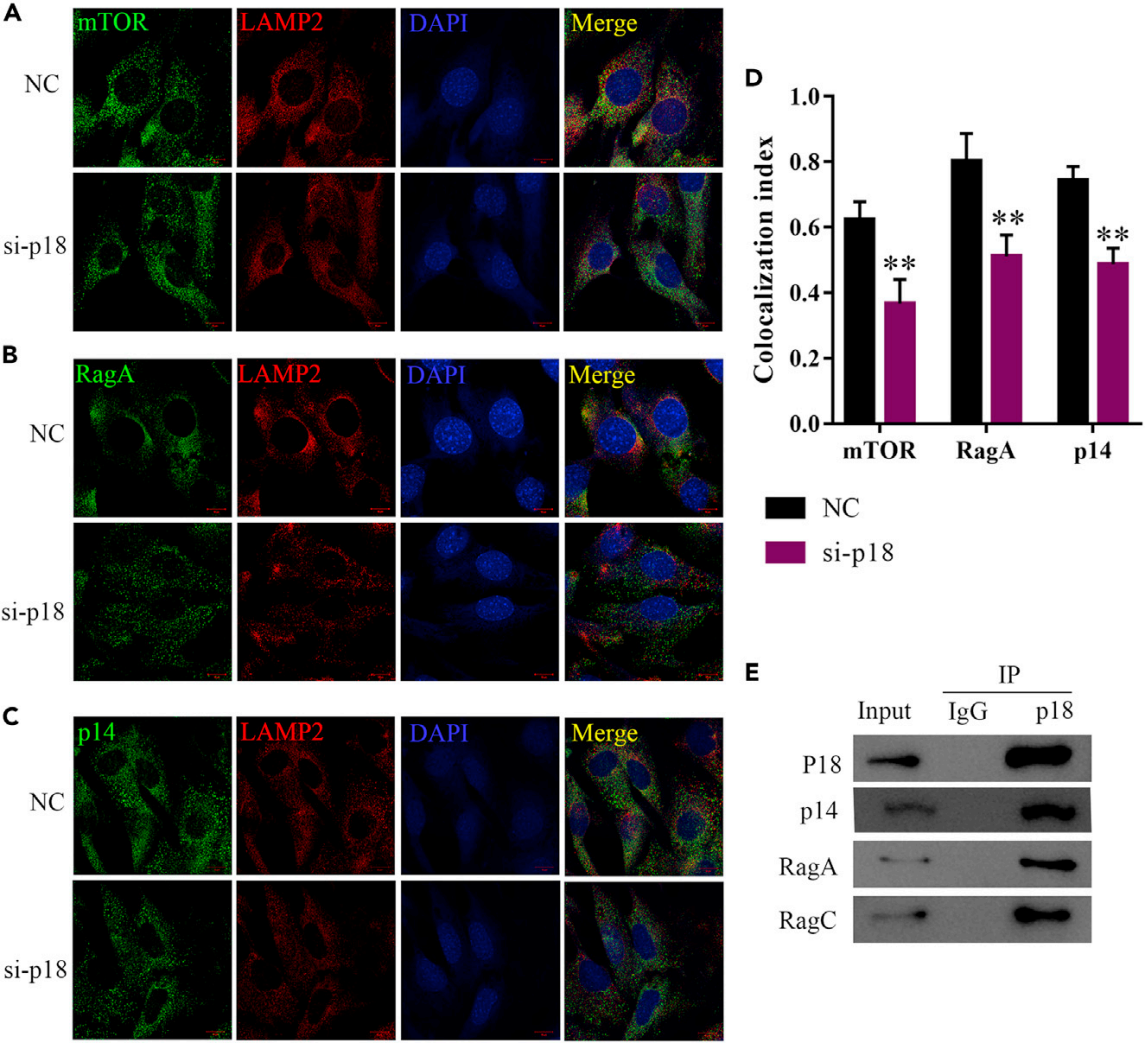
Increased p18 levels are associated with increased lysosomal localization of the Ragulator-Rag complex and mTORC1 (A) Images of GBC-SD cells co-immunostained for lysosomal protein LAMP2 (red) and mTOR (green). (B) Images of GBC-SD cells co-immunostained for lysosomal protein LAMP2 (red) and RagA (green). (C) Images of GBC-SD cells co-immunostained for lysosomal protein LAMP2 (red) and p14 (green). (D) Quantification of mTOR-LAMP2, RagA-LAMP2, and p14-LAMP2 colocalization in GBC-SD cells. (E) p18 co-immunoprecipitates RagA, RagC, and p14 in GBC-SD cells. ∗∗*p* < 0.01.

To confirm whether increased p18 levels are associated with the increased lysosomal localization of the Ragulator-Rag complex and mTORC1, we used fluorescent imaging of co-immunostained lysosomal protein LAMP2 and either mTOR, RagA, or p14 with or without the expression of p18 (Figures 7A–7C). The colocalization index of mTOR-LAMP2, RagA-LAMP2, and p14-LAMP2 was significantly higher when p18 was present (Figure 7D). Further analysis using co-IP demonstrated that p18 interacts with RagA, RagC, and p14 in GBC-SD cells (Figure 7E). These results confirm that increased p18 levels facilitate lysosomal anchoring of the Ragulator-Rag complex and activation of mTORC1.

### Inhibiting p18 counteracts abnormal mTORC1 signaling induced by RNF152 deficiency

**Figure 8:**
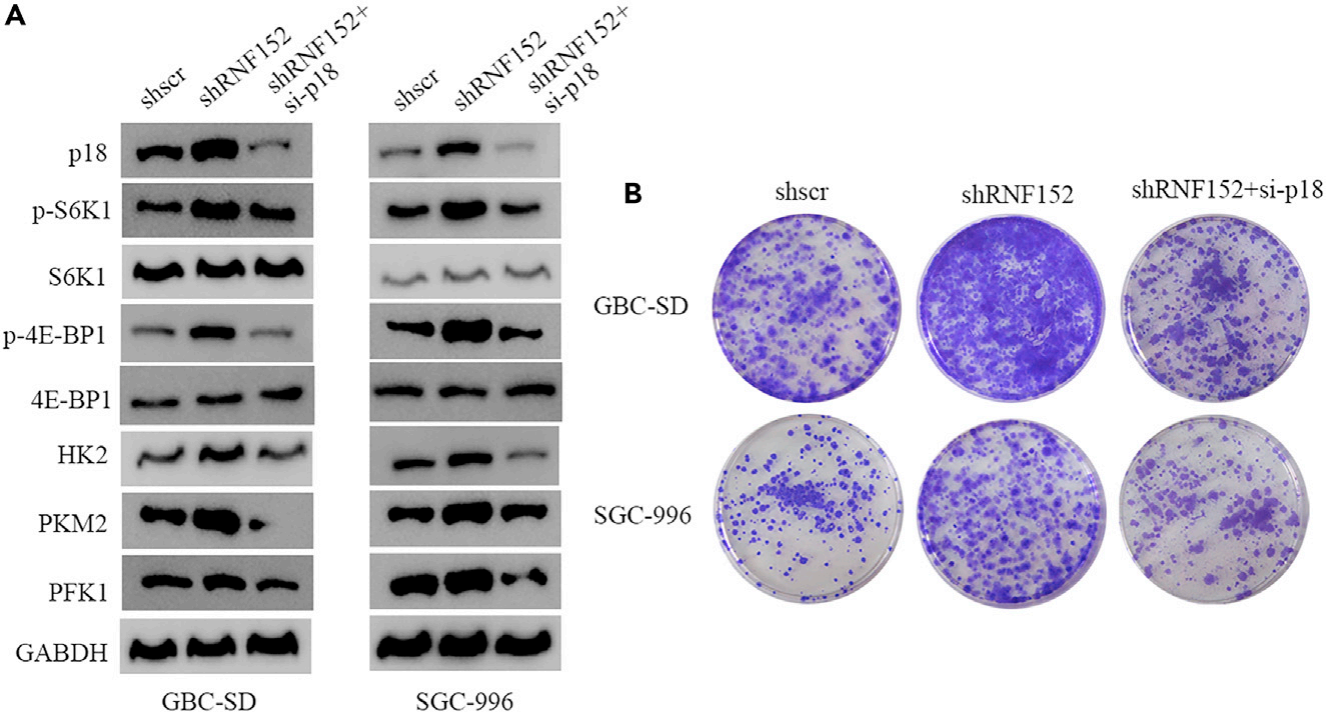
Knockdown of p18 counteracts RNF152 deficiency-induced abnormal mTORC1 signaling (A) Western blotting experiments to assess the expression of RNF152, p18, mTORC1 substrate, and rate-limiting glycolytic enzymes in GBC-SD and SGC-996 cells. (B) Knockdown of p18 reversed the promotive effects of RNF152 deficiency in colony formation in GBC-SD and SGC-996 cells.

We next determined whether the knockdown of p18 could influence mTORC1 in a way that is opposite to the effects of RNF152 deficiency. We measured the expression of RNF152, p18, mTORC1 substrate, and rate-limiting glycolytic enzymes in GBC-SD and SGC-996 cells by western blotting and found that p18 knockdown could reverse the effects of RNF152 deficiency (Figure 8A). These results were verified by colony formation in GBC-SD and SGC-996 cells (Figure 8B). The knockdown of p18 reduced the high levels of colonies formed when RNF152 was inhibited. The silencing of both RNF152 and p18 led to lower levels of cell migration. This would imply that although p18 is not required for mitochondrial oxidative phosphorylation under fasting conditions, it is important in the glycolysis and proliferation of GBC cells.

### RNF152 enhanced the sensitivity of GEM by inhibiting mTORC1 signaling and glycolysis *in vivo*

**Figure 9:**
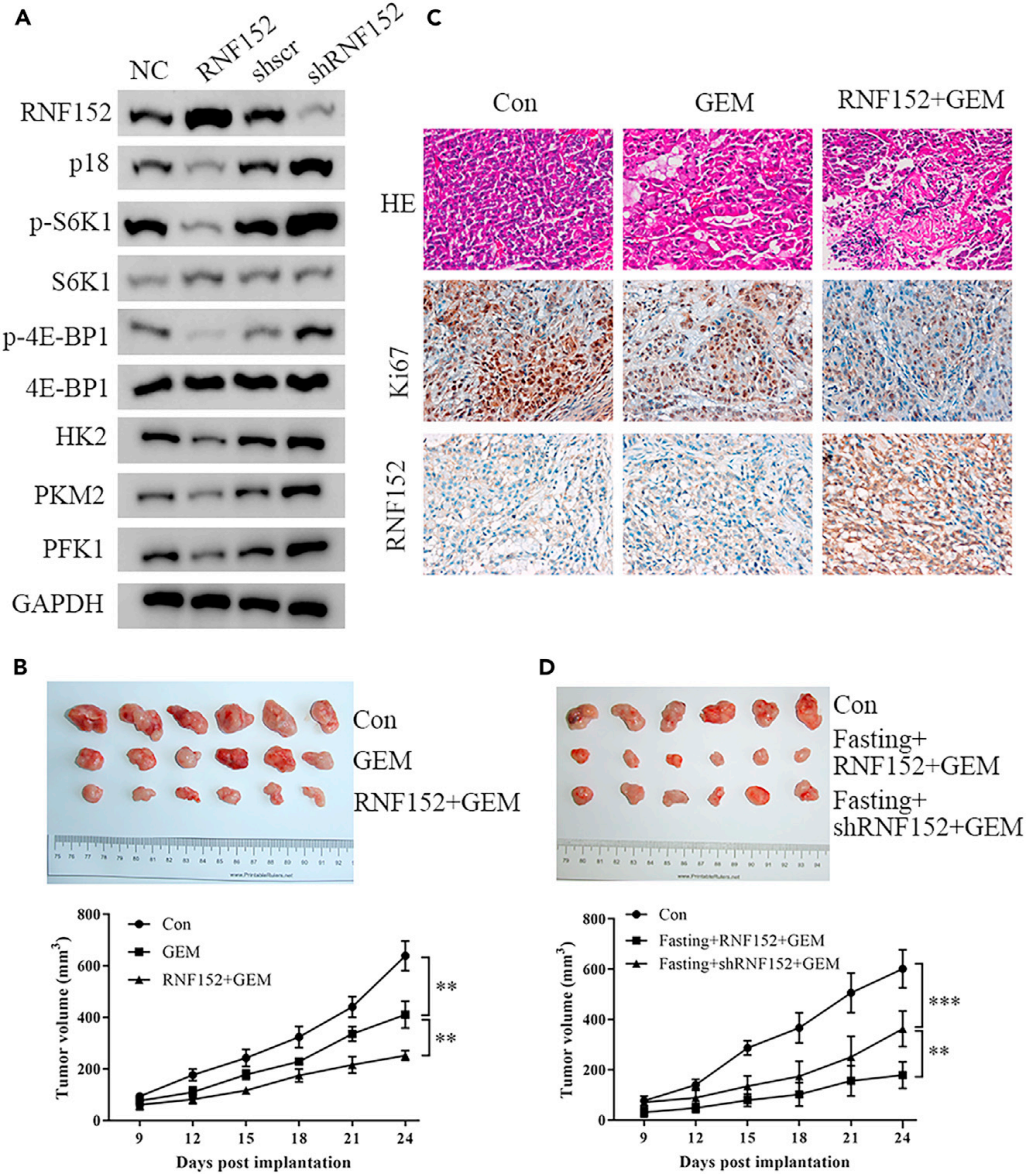
RNF152 enhanced the sensitivity of gemcitabine by inhibiting mTORC1 signaling and glycolysis (A) Western blot analysis of xenograft tumors from BALB/c nude mice subcutaneously injected with transfected GBC-SD and SGC-996 cells for the expression of RNF152, p18, mTORC1 substrate, and rate-limiting glycolytic enzymes. (B) *In vivo* growth of tumors as measured by tumor volume in mice inoculated with GBC-SD cells; Images and volume of tumors isolated from mice in different groups. (C) Representative HE and immunohistochemistry staining for the expressions of Ki-67 and RNF152. Scale bar, 50 μm. (D) Images and volume of tumors isolated from mice in different groups. ∗∗*p* < 0.01, ∗∗∗*p* < 0.001.

Having established that RNF152 enhanced the sensitivity of GEM through inhibiting mTORC1 signaling and glycolysis in GBC cells, we investigated whether similar results could be obtained *in vivo*. GBC cells under or overexpressing RNF152 were transplanted in mice to create a xenograft model. After 24 days the tumors were assessed for the expression of RNF152, p18, mTORC1 substrate, and rate-limiting glycolytic enzymes (Figure 9A). The overexpression of RNF152 led to reduced levels of p18 and other proteins associated with glycolysis. However, the loss of RNF152 expression had the opposite effect and increased levels of glycolytic enzymes. When mice were treated with GEM, the tumors overexpressing RNF152 were significantly smaller than the control tumors (Figure 9B). Representative tumor samples stained for the expressions of Ki-67 indicate that the overexpression of RNF152 results in a lower level of cell proliferation and increased sensitivity to GEM (Figure 9C). Tumor volume indicates that fasting can enhance sensitivity to GEM, however when the expression of RNF152 is inhibited in tumors, the resistance to GEM is increased (Figure 9D). With the collation of these results, we can conclude that the upregulation of RNF152 through fasting can enhance the sensitivity of GBC cells to GEM through inhibiting mTORC1 signaling and glycolysis.

## Discussion

The survival rate of patients with GBC remains a concern because it is diagnosed at a late stage when metastasis and drug resistance have developed.^5^^,^^33^ Therefore, a key factor in improving the therapeutic outcome of GBC is through the enhancement of drug sensitivity by using a combination of therapies.^4^ Intermittent fasting is gaining credibility as a method of enhancing the chemotherapeutic susceptibility of tumors.^34^^,^^35^ However, the exact metabolic pathways involved and how they affect cancer cells are unclear. Therefore, in this study, we examined the effects of fasting on RNF152, a ubiquitin ligase that is downregulated in several biliary tract cancers.^27^ Our research suggests that RNF152 can act as a fasting sensor and is an important regulator of the p18/mTORC1 pathway in GBC cells. RNF152 suppresses the activity of mTORC1 in GBC cells to inhibit glycolysis and enhance the sensitivity of GBC cells to GEM.

In our study, RNF152 was downregulated in the tumor tissue of GBC patients and in proliferating GBC cells. However, the overexpression of RNF152 led to a reduction in the proliferation of GBC cells. Moreover, fasting was found to increase the expression of RNF152. RNF152 is known to be downregulated in other cancers, including hepatocellular carcinoma and colorectal cancer (CRC).^26^^,^^27^^,^^36^ Increasing evidence suggests that RNF152 can suppress the proliferation of cancer cells by regulating mTORC1 through a mechanism involving ubiquitination. Deng et al. discovered that RNF152 negatively regulates mTORC1 by targeting RagA for K63-linked ubiquitination.^28^ In another study, RagA was not ubiquitylated in CRC cells whereas mTORC1 was upregulated.^27^ An interesting finding in our study was that levels of RNF152 could be controlled through fasting. In support of this finding, Weng et al. discovered that fasting could inhibit aerobic glycolysis and proliferation in CRC by the suppression of the AKT/mTOR/HIF1α pathway.^18^ Similarly, we have found that fasting can inhibit this pathway in GBC through RNF152 ubiquitination.

Several cancers are associated with the aberrant regulation of p18, including metastatic melanoma, bladder cancer, and hepatocellular carcinoma.^30^^,^^37^^,^^38^ In our study, p18 contributed to drug resistance and glycolysis by modulating the location and lysosomal activity of the Ragulator-Rag complex formed with mTORC1. We found that inhibiting p18 counteracts abnormal mTORC1 signaling induced by RNF152 deficiency. Similar mechanisms of drug resistance involving the combination of RNF152 and the p18/mTORC1 pathway have been reported.^30^^,^^31^ The upregulation of RNF152 is known to control levels of mTORC1 through the K63-linked ubiquitination of RagA thereby preventing the assembly of the p18 and Ragulator-Rag complex.^28^ Moreover, the ubiquitin E3 ligase UBE3A has been found to regulate mTORC1 activity by targeting p18/LAMTOR1.^39^ Sun et al. discovered that ubiquitination and degradation of p18 by UBE3A results in decreased mTORC1 activity and UBE3A knockdown results in increased lysosomal localization of p18 and other members of the Ragulator-Rag complex.^39^ Furthermore, the upregulation of mTORC1 by other factors is known to increase glycolysis and is associated with cancer. For instance, the interaction of galectin-3 with Rag GTPases and Ragulator on lysosomes leads to the promotion of glycolysis through the activation of mTORC1.^40^ Galectin-3 is upregulated in several cancers and is believed to be associated with glycolysis and mitochondrial metabolism in tumors.^41^^,^^42^ However, whether galectin-3 or UBE3A form part of a network with RNF152 in regulating the Ragulator-Rag complex and p18/mTORC1 pathway remains to be investigated. Certainly, the relationship between p18/mTORC1, metabolic pathways, and drug resistance has been well-established and our results support these findings.^43^^,^^44^

There are limitations to our study, including the low number of tissue samples examined and the inability to monitor the calorific intake or the physiological and metabolic status of patients with GBC. In addition, ethnicity was restricted to a limited genetic background, and further studies of RNF152 expression in other genetic backgrounds are required. However, the uniqueness of this study lies in the ability to associate metabolic pathways with the differential expression of RNF152 and the activation of the p18/mTORC1 pathway. To conclude, our research suggests that the expression of RNF152 can be enhanced through fasting and can mediate the metabolic status of GBC cells. Moreover, RNF152 is an important regulator of mTORC1 signaling by mediating the degradation of p18. Therefore, RNF152 functions as a tumor suppressor protein by inhibiting the activity of mTORC1 to suppress glycolysis and enhance the sensitivity of GEM in GBC. Further investigations could uncover other components involved in the RNF152, p18, and mTORC1 network and whether this could be manipulated by fasting. Manipulating the expression of RNF152 may have potential in the management of GBC.

## STAR★Methods

### Key resources table

**Table:** 

REAGENT or RESOURCE	SOURCE	IDENTIFIER
**Antibodies**		

Anti-RNF152	Santa Cruz Biotechnology	Cat#sc-398407
Anti-p18	Cell Signaling Technology	Cat#8975
Anti-p-S6K1 Thr389	Cell Signaling Technology	Cat#9205
Anti-S6K1	Cell Signaling Technology	Cat#9202
Anti-p-4E-BP1 Ser65	Cell Signaling Technology	Cat#9451
Anti-4E-BP1	Cell Signaling Technology	Cat#9644
Anti-HK2	Proteintech	Cat#22029-1-AP
Anti-PKM2	Proteintech	Cat#60268-1-Ig
Anti-PFK1	Santa Cruz Biotechnology	Cat#sc-377346

**Biological samples**		

Human GBC tissues, and adjacent normal tissues	Shanghai Xinhua Hospital	N/A

**Chemicals, peptides, and recombinant proteins**		

Fetal bovine serum	GIBCO	Cat#A5669701
RIPA buffe	Sigma-Aldrich	Cat#R0278
BCA Reagent	Thermo Fisher Scientific	Cat#A55861
DAPI	Sigma-Aldrich	Cat#17510
Gemcitabine	MedChem Express	Cat#HY-17026
Lipofectamine 3000	Invitrogen	Cat#L3000001

**Critical commercial assays**		

Cell Counting Kit-8	Dojindo	Cat#CK04
Click-iT EdU Imaging Kit	Invitrogen	Cat#C10339
Annexin-V/PI apoptosis kit	MultiSciences	Cat#70-AT107-30
Glucose Uptake Fluorometric Assay Kit	Biovision	Cat##K666-100
Lactate Colorimetric Assay Kit	Biovision	Cat##K627-100
Seahorse XF Cell Mito Stress Test Kits	Seahorse Bioscience	Cat#103015-100
Seahorse XF Glycolysis Stress Test Kits	Seahorse Bioscience	Cat#103020-100

**Deposited data**		

Unbiased genome-wide CRISPR-Cas9 screen	Xu et al.^25^	Xu et al.^25^

**Experimental models: Cell lines**		

GBC-SD	Chinese Academy of Sciences	N/A
SGC-996	Chinese Academy of Sciences	N/A

**Experimental models: Organisms/strains**		

Mouse: BALB/c nude	Vitalriver	N/A

**Oligonucleotides**		

RNF152 shRNA: 5′-CCGGATGTCAGATCTGTTTCAATTAC TCGAGTAATTGAAACAGATCTGACATTTTTTTG-3′	GenePharma	N/A
p18 siRNA: 5′-GGAGCUGGUUGUACAGUUU-3′	GenePharma	N/A

**Software and algorithms**		

ImageJ Software	National Institutes of Health	https://imagej.nih.gov/ij
GraphPad Prism 6.0	GraphPad Prism Software	https://www.graphpad.com/
SPSS 22.0 statistical software	IBM Corp	https://www.ibm.com/products/spss-statistics

### Resource availability

#### Lead contact

Requests for further information or materials should be directed to and will be fulfilled by the lead contact, Tao suo (suo.tao@zs-hospital.sh.cn).

#### Materials availability

This study did not generate any new unique reagents.

#### Data and code availability

• All data reported in this paper can be made available by lead contact upon request.
• This paper does not report original code.
• Any additional information required to reanalyze the data reported in this paper is available from the lead contact upon request.

### Experimental model and study participant details

#### Cell lines and tissues

The human GBC cell lines (GBC-SD and SGC-996) were purchased from the Cell Bank of Type Culture Collection of the Chinese Academy of Sciences (Beijing, China). Cells were maintained in Dulbecco’s modified Eagle medium (DMEM) supplemented with 10% fetal bovine serum (FBS, Thermo Fisher Scientific, Waltham, MA, USA) at 37°C in a humidified atmosphere with a humidified atmosphere containing 5% CO_2_. Fasting cells were cultured under the same conditions in a fasting mimic medium (glucose-free DMEM supplemented with 0.5 g/L glucose and 1% FBS) for 48 h, as described previously.^18^

Clinical cancer tissues and their paired adjacent normal tissues were collected from GBC patients (*N* = 25) in Zhongshan Hospital, Fudan University, Shanghai, China. Ethical approval for the use of human subjects was obtained from the Research Ethics Committee of Zhongshan Hospital consistent with ethical guidelines of the 1975 Declaration of Helsinki (B2022-364R), and informed consent was obtained from each patient. The information on clinical characteristics and clinical outcomes is presented in Table 1. The clinical and demographic information of gallbladder cancer patients is shown in Table S1.

#### Mice and ethics statement

Ethical approval was obtained from the Zhongshan Hospital Research Ethics Committee consistent with ethical guidelines of the 1975 Declaration of Helsinki, Animal Ethics Approval is shown in Figure S1. BALB/c male nude mice (4–5 weeks of age, weighing 18–20 g) were purchased from Vitalriver (Beijing, China) and housed in pathogen-free conditions.

### Method details

#### Cell transfection

Lentivirus encoding RNF152 shRNA (shRNF152) and a nontargeting shRNA (shNC) were purchased from GenePharma (Suzhou, China). The sequence of the RNF152-shRNA was 5′-CCGGATGTCAGATCTGTTTCAATTACTCGAGTAATTGAAACAGATCTGACATTTTTTTG-3′. The siRNAs targeting p18 (si-p18) and control siRNA were obtained from GenePharma. The sequence of the si-p18 was 5′-GGAGCUGGUUGUACAGUUU-3′. To overexpress RNF152, pEnter-RNF152 was purchased from Vigene Biosciences (Shandong, China). Cell transfection with plasmids or siRNAs was conducted using Lipofectamine 3000 (Invitrogen, Waltham, MA, USA) following the manufacturer’s instructions. An RNF152 cell clone overexpressing RNF152 was established using the retroviral vector pMY-IG as described previously.^45^

#### Western blotting, immunohistochemistry (IHC), and co-immunoprecipitation

Protein was extracted from cells using RIPA lysis buffer (Sigma-Aldrich, St. Louis, MO, USA). Protein concentration was determined with BCA Reagent (Thermo Fisher Scientific) and equal amounts of protein were separated using 10% SDS-PAGE. The proteins were transferred to a polyvinylidene difluoride membrane blocked in low-fat milk and incubated with primary antibodies overnight at 4°C. The primary antibodies used in this study were RNF152 (sc-398407, Santa Cruz Biotechnology, Dallas, TX, USA), p18 (Cat#8975, Cell Signaling Technology, Danvers, MA, USA), p-S6K1 Thr389 (Cat#9205, Cell Signaling Technology), S6K1 (Cat#9202, Cell Signaling Technology), p-4E-BP1 Ser65 (Cat#9451, Cell Signaling Technology), 4E-BP1 (Cat#9644, Cell Signaling Technology), HK2 (22029-1-AP, Proteintech, Rosemont, IL, USA), PKM2 (60268-1-Ig, Proteintech), PFK1 (Cat#sc-377346, Santa Cruz Biotechnology), and GAPDH (sc-365,062, Santa Cruz Biotechnology). The membranes were incubated with secondary antibodies (Boster, Wuhan, China) for 1 h and then immunoreactive bands were detected with enhanced chemiluminescence reagent (Boster).

IHC analysis was performed as described previously using antibodies toward Ki67 (273019-I-AP, ProteinTech) and RNF152 (sc-398407, Santa Cruz Biotechnology).^46^ The sections were scored by two independent reviewers using a scale from 0 to 4 for the proportion of tumor cells (0, no positive tumor cells; 1, <10% positive tumor cells; 2, 10–50% positive tumor cells; 3, 50–75% positive tumor cells; and 4, >75% positive tumor cells) and a scale from 1 to 3 for the intensity of the staining (1, no staining; 2, weak staining; and 3, strong staining). The staining index was determined as the proportion of tumor cells × staining score.

Co-immunoprecipitation (IP) experiments were performed using antibodies toward RagC (Cat#5466, Cell Signaling Technology) as described previously.^47^ Briefly, cells were lysed with RIPA buffer and then centrifuged at full speed for 15 min to obtain the supernatant, which was incubated with the RagC antibody, IgG, and protein G agarose (Pierce, Rockford, IL, USA) at 4°C overnight. The beads were washed with IP buffer and proteins were eluted by heating the samples at 100°C for 5 min. The eluted proteins were separated with SDS-PAGE and analyzed by western blotting.

#### *In vitro* ubiquitination assay

The *in vitro* ubiquitination of p18 was measured using a Ubiquitin Ligase Kit (Boston Biochem, Cambridge, MA, USA) according to the manufacturer’s instructions. Samples were separated by SDS-PAGE and subjected to western blotting. Ubiquitination was detected by incubating western blots with p18 and ubiquitin antibodies (Cell Signaling Technology).

#### Cell proliferation and apoptosis assay

Following each treatment, cell proliferation and viability were measured by using a Cell Counting Kit-8 (CCK8, Dojindo Laboratories, Kumamoto, Japan) according to the manufacturer’s instructions and EdU staining (Click-iT EdU Imaging Kit, #C10339, Invitrogen). Cells stained with CCK-8 reagent were incubated at 37°C for 2 h and then measured at an absorbance of 450 nm. To determine apoptosis, cells were stained using an Annexin-V/PI apoptosis kit (MultiSciences, Hangzhou, China) according to the manufacturer’s instructions. Then Annexin-V+ cells were examined by flow cytometry (BD Biosciences, San Jose, CA, USA).

#### Colony formation and cell migration

Following treatment, cells (500 cells/well) were seeded into six-well plates. After 2 weeks, the cells were fixed and stained with 0.05% Crystal Violet and the number of colonies was counted by light microscopy. To determine the migration of cells we performed a wound-healing assay. Cells were grown to confluence in six-well plates, a scratch was made on the surface of each well with a pipette tip. Images of migration into the scratch were recorded after 24 h.

#### OCR, ECAR, and glycolysis assay

The mitochondrial function and glycolytic capacity of GBC cells were measured using a Seahorse Bioscience XF96 Extracellular Flux Analyzer and Seahorse XF Glycolysis Stress and Cell Mito Stress Test Kits (Seahorse Bioscience, North Billerica, MA, USA) according to the manufacturer’s instructions. The OCR and ECAR values were normalized to the total cell number and presented as the mean ± SD. Glycolysis was measured through glucose uptake and lactate production by using a Glucose Uptake Fluorometric Assay Kit (Biovision, Waltham, MA, USA; #K666-100) and a Lactate Colorimetric Assay Kit (Biovision, #K627-100), respectively.

#### Immunofluorescence staining

Cells were fixed in 4% paraformaldehyde, permeabilized with 0.2% Triton X-100, and blocked with 0.8% bovine serum albumin. They were then incubated with appropriate primary antibodies, mTOR (Cat#2983, Cell Signaling Technology), LAMP2 (ab13524, Abcam), RagA (Cat#4357, Cell Signaling Technology), or p14 (Cat#8975, Cell Signaling Technology), overnight at 4°C. Then they were incubated with Alexa Fluor secondary antibodies (Invitrogen) and nuclei were stained with DAPI (Sigma-Aldrich). Images were visualized with an Olympus (Tokyo, Japan) inverted microscope.

#### Tumor xenografts

GBC cells (2 × 10^4^) overexpressing or inhibiting RNF152 were injected subcutaneously into the right flanks of the mice and the size of the tumors was monitored weekly. Mice were randomly assigned to control and fasting groups (*n* = 6 per group). The control mice were fed rodent chow *ad libitum* (average daily consumption was 15 kJ) whereas the fasting groups were fed a 7-day diet that contained 50% of the normal calorific intake on day 1, 10% of the normal calorific intake on days 2 and 3, and the normal calorific intake on days 4–7. This cycle was repeated. All animals had free access to water. At the end of the experiment, the mice were killed humanely and the tumors were dissected. Tumor volume was calculated by the formula: volume = (length × width^2^)/2.

### Quantification and statistical analysis

All statistical analyses were performed by using SPSS 22.0 software (IBM Corp., Armonk, NY, USA) and presented as an average of biological replicates (mean ± S.D.). Student’s t test or one-way ANOVA was used to evaluate the differences. All graphs were generated using GraphPad Prism 6.0 software (GraphPad Software Inc., La Jolla, CA, USA). *p* < 0.05 was considered statistically significant.

## Supplemental information

Document S1. Figures S1

Table S1. Clinical and demographic information of gallbladder cancer patients

## References

[1] Roa J.C., García P., Kapoor V.K., Maithel S.K., Javle M., Koshiol J. (2022). Gallbladder cancer. Nat. Rev. Dis. Primers.

[2] Schmidt M.A., Marcano-Bonilla L., Roberts L.R. (2019). Gallbladder cancer: epidemiology and genetic risk associations. Chin. Clin. Oncol..

[3] Valle J.W., Kelley R.K., Nervi B., Oh D.Y., Zhu A.X. (2021). Biliary tract cancer. Lancet.

[4] Harding J.J., Khalil D.N., Fabris L., Abou-Alfa G.K. (2023). Rational development of combination therapies for biliary tract cancers. J. Hepatol..

[5] Mayr C., Kiesslich T., Modest D.P., Stintzing S., Ocker M., Neureiter D. (2022). Chemoresistance and resistance to targeted therapies in biliary tract cancer: what have we learned?. Expert Opin. Investig. Drugs.

[6] Gonçalves A.C., Richiardone E., Jorge J., Polónia B., Xavier C.P.R., Salaroglio I.C., Riganti C., Vasconcelos M.H., Corbet C., Sarmento-Ribeiro A.B. (2021). Impact of cancer metabolism on therapy resistance - Clinical implications. Drug Resist. Updat..

[7] Infantino V., Santarsiero A., Convertini P., Todisco S., Iacobazzi V. (2021). Cancer cell metabolism in hypoxia: Role of HIF-1 as key regulator and therapeutic target. Int. J. Mol. Sci..

[8] Park J.H., Pyun W.Y., Park H.W. (2020). Cancer metabolism: phenotype, signaling and therapeutic targets. Cells.

[9] Abdel-Wahab A.F., Mahmoud W., Al-Harizy R.M. (2019). Targeting glucose metabolism to suppress cancer progression: prospective of anti-glycolytic cancer therapy. Pharmacol. Res..

[10] Icard P., Shulman S., Farhat D., Steyaert J.M., Alifano M., Lincet H. (2018). How the Warburg effect supports aggressiveness and drug resistance of cancer cells?. Drug Resist. Updat..

[11] Vaupel P., Schmidberger H., Mayer A. (2019). The Warburg effect: essential part of metabolic reprogramming and central contributor to cancer progression. Int. J. Radiat. Biol..

[12] He Y., Chen X., Yu Y., Li J., Hu Q., Xue C., Chen J., Shen S., Luo Y., Ren F. (2018). LDHA is a direct target of miR-30d-5p and contributes to aggressive progression of gallbladder carcinoma. Mol. Carcinog..

[13] Zheng P., Wang X., Hong Z., Shen F., Zhang Q. (2019). Preoperative fasting hyperglycemia is an independent prognostic factor for postoperative survival after gallbladder carcinoma radical surgery. Cancer Manag. Res..

[14] Poff A., Koutnik A.P., Egan K.M., Sahebjam S., D'Agostino D., Kumar N.B. (2019). Targeting the Warburg effect for cancer treatment: Ketogenic diets for management of glioma. Semin. Cancer Biol..

[15] Zhang J., Deng Y., Khoo B.L. (2020). Fasting to enhance Cancer treatment in models: the next steps. J. Biomed. Sci..

[16] Mercier B.D., Tizpa E., Philip E.J., Feng Q., Huang Z., Thomas R.M., Pal S.K., Dorff T.B., Li Y.R. (2022). Dietary Interventions in cancer treatment and response: A comprehensive review. Cancers.

[17] Simone B.A., Champ C.E., Rosenberg A.L., Berger A.C., Monti D.A., Dicker A.P., Simone N.L. (2013). Selectively starving cancer cells through dietary manipulation: methods and clinical implications. Future Oncol..

[18] Weng M.L., Chen W.K., Chen X.Y., Lu H., Sun Z.R., Yu Q., Sun P.F., Xu Y.J., Zhu M.M., Jiang N. (2020). Fasting inhibits aerobic glycolysis and proliferation in colorectal cancer via the Fdft1-mediated AKT/mTOR/HIF1alpha pathway suppression. Nat. Commun..

[19] Nencioni A., Caffa I., Cortellino S., Longo V.D. (2018). Fasting and cancer: molecular mechanisms and clinical application. Nat. Rev. Cancer.

[20] Krstic J., Reinisch I., Schindlmaier K., Galhuber M., Riahi Z., Berger N., Kupper N., Moyschewitz E., Auer M., Michenthaler H. (2022). Fasting improves therapeutic response in hepatocellular carcinoma through p53-dependent metabolic synergism. Sci. Adv..

[21] Raffaghello L., Lee C., Safdie F.M., Wei M., Madia F., Bianchi G., Longo V.D. (2008). Starvation-dependent differential stress resistance protects normal but not cancer cells against high-dose chemotherapy. Proc. Natl. Acad. Sci. USA.

[22] Omar E.M., Omran G.A., Mustafa M.F., El-Khodary N.M. (2022). Intermittent fasting during adjuvant chemotherapy may promote differential stress resistance in breast cancer patients. J. Egypt. Natl. Canc. Inst..

[23] Cai C., Tang Y.D., Zhai J., Zheng C. (2022). The RING finger protein family in health and disease. Signal Transduct. Target. Ther..

[24] Huang N., Sun X., Li P., Liu X., Zhang X., Chen Q., Xin H. (2022). TRIM family contribute to tumorigenesis, cancer development, and drug resistance. Exp. Hematol. Oncol..

[25] Xu S., Zhan M., Jiang C., He M., Yang L., Shen H., Huang S., Huang X., Lin R., Shi Y. (2019). Genome-wide CRISPR screen identifies ELP5 as a determinant of gemcitabine sensitivity in gallbladder cancer. Nat. Commun..

[26] Wan J., Liu S., Sun W., Yu H., Tang W., Liu W., Ji J., Liu B. (2021). Ring finger protein 152-dependent degradation of TSPAN12 suppresses hepatocellular carcinoma progression. Cancer Cell Int..

[27] Cui X., Shen W., Wang G., Huang Z., Wen D., Yang Y., Liu Y., Cui L. (2018). Ring finger protein 152 inhibits colorectal cancer cell growth and is a novel prognostic biomarker. Am. J. Transl. Res..

[28] Deng L., Jiang C., Chen L., Jin J., Wei J., Zhao L., Chen M., Pan W., Xu Y., Chu H. (2015). The ubiquitination of rag A GTPase by RNF152 negatively regulates mTORC1 activation. Mol. Cell.

[29] Wu W.D., Hu Z.M., Shang M.J., Zhao D.J., Zhang C.W., Hong D.F., Huang D.S. (2014). Cordycepin down-regulates multiple drug resistant (MDR)/HIF-1alpha through regulating AMPK/mTORC1 signaling in GBC-SD gallbladder cancer cells. Int. J. Mol. Sci..

[30] Palušová V., Renzová T., Verlande A., Vaclová T., Medková M., Cetlová L., Sedláčková M., Hříbková H., Slaninová I., Krutá M. (2020). Dual targeting of BRAF and mTOR signaling in melanoma cells with pyridinyl imidazole compounds. Cancers.

[31] Szwed A., Kim E., Jacinto E. (2021). Regulation and metabolic functions of mTORC1 and mTORC2. Physiol. Rev..

[32] Dodd K.M., Yang J., Shen M.H., Sampson J.R., Tee A.R. (2015). mTORC1 drives HIF-1α and VEGF-A signalling via multiple mechanisms involving 4E-BP1, S6K1 and STAT3. Oncogene.

[33] Yang L., Shi Y.L., Ma Y., Ren W.W., Pang G.M., Liu J. (2022). Silencing KLF16 inhibits oral squamous cell carcinoma cell proliferation by arresting the cell cycle and inducing apoptosis. APMIS.

[34] Clifton K.K., Ma C.X., Fontana L., Peterson L.L. (2021). Intermittent fasting in the prevention and treatment of cancer. CA. Cancer J. Clin..

[35] Sadeghian M., Rahmani S., Khalesi S., Hejazi E. (2021). A review of fasting effects on the response of cancer to chemotherapy. Clin. Nutr..

[36] Okamoto T., Imaizumi K., Kaneko M. (2020). The role of tissue-specific ubiquitin ligases, RNF183, RNF186, RNF182 and RNF152, in disease and biological function. Int. J. Mol. Sci..

[37] Sun Y., Guan Z., Sheng Q., Duan W., Zhao H., Zhou J., Deng Q., Pei X. (2022). N-myristoyltransferase-1 deficiency blocks myristoylation of LAMTOR1 and inhibits bladder cancer progression. Cancer Lett..

[38] Wu B., Wang Q., Li B., Jiang M. (2022). LAMTOR1 degrades MHC-II via the endocytic in hepatocellular carcinoma. Carcinogenesis.

[39] Sun J., Liu Y., Jia Y., Hao X., Lin W.J., Tran J., Lynch G., Baudry M., Bi X. (2018). UBE3A-mediated p18/LAMTOR1 ubiquitination and degradation regulate mTORC1 activity and synaptic plasticity. Elife.

[40] Chen X., Yu C., Liu X., Liu B., Wu X., Wu J., Yan D., Han L., Tang Z., Yuan X. (2022). Intracellular galectin-3 is a lipopolysaccharide sensor that promotes glycolysis through mTORC1 activation. Nat. Commun..

[41] Guo Y., Shen R., Yu L., Zheng X., Cui R., Song Y., Wang D. (2020). Roles of galectin-3 in the tumor microenvironment and tumor metabolism (Review). Oncol. Rep..

[42] Li Y.S., Li X.T., Yu L.G., Wang L., Shi Z.Y., Guo X.L. (2020). Roles of galectin-3 in metabolic disorders and tumor cell metabolism. Int. J. Biol. Macromol..

[43] Murugan A.K. (2019). mTOR: Role in cancer, metastasis and drug resistance. Semin. Cancer Biol..

[44] Hua H., Kong Q., Zhang H., Wang J., Luo T., Jiang Y. (2019). Targeting mTOR for cancer therapy. J. Hem. Oncol..

[45] Tanaka K., Ikeda N., Miyashita K., Nuriya H., Hara T. (2018). DEAD box protein DDX1 promotes colorectal tumorigenesis through transcriptional activation of the LGR5 gene. Cancer Sci..

[46] Xia J., Wu Z., Yu C., He W., Zheng H., He Y., Jian W., Chen L., Zhang L., Li W. (2012). miR-124 inhibits cell proliferation in gastric cancer through down-regulation of SPHK1. J. Pathol..

[47] Fu Z., Liang X., Shi L., Tang L., Chen D., Liu A., Shao C. (2021). SYT8 promotes pancreatic cancer progression via the TNNI2/ERRα/SIRT1 signaling pathway. Cell Death Discov..

