# Supplementary material for: Fasting-induced RNF152 resensitizes gallbladder cancer cells to gemcitabine by inhibiting mTORC1-mediated glycolysis
Source: iScience. 2024 Apr 8;27(5):109659. doi: 10.1016/j.isci.2024.109659 (PMC11068552; doi:10.1016/j.isci.2024.109659)
Supplement: Document S1. Figures S1 [file mmc1.pdf]

iScience, Volume *27*

Supplemental information

Fasting-induced RNF152 resensitizes gallbladder cancer cells to gemcitabine by inhibiting mTORC1-mediated glycolysis

Ying Tao, Zijun Gong, Sheng Shen, Yaqi Ding, Rui Zan, Bohao Zheng, Wentao Sun, Chaolin Ma, Mengxuan Shu, Xiao Lu, Han Liu, Xiaoling Ni, Houbao Liu, and Tao Suo

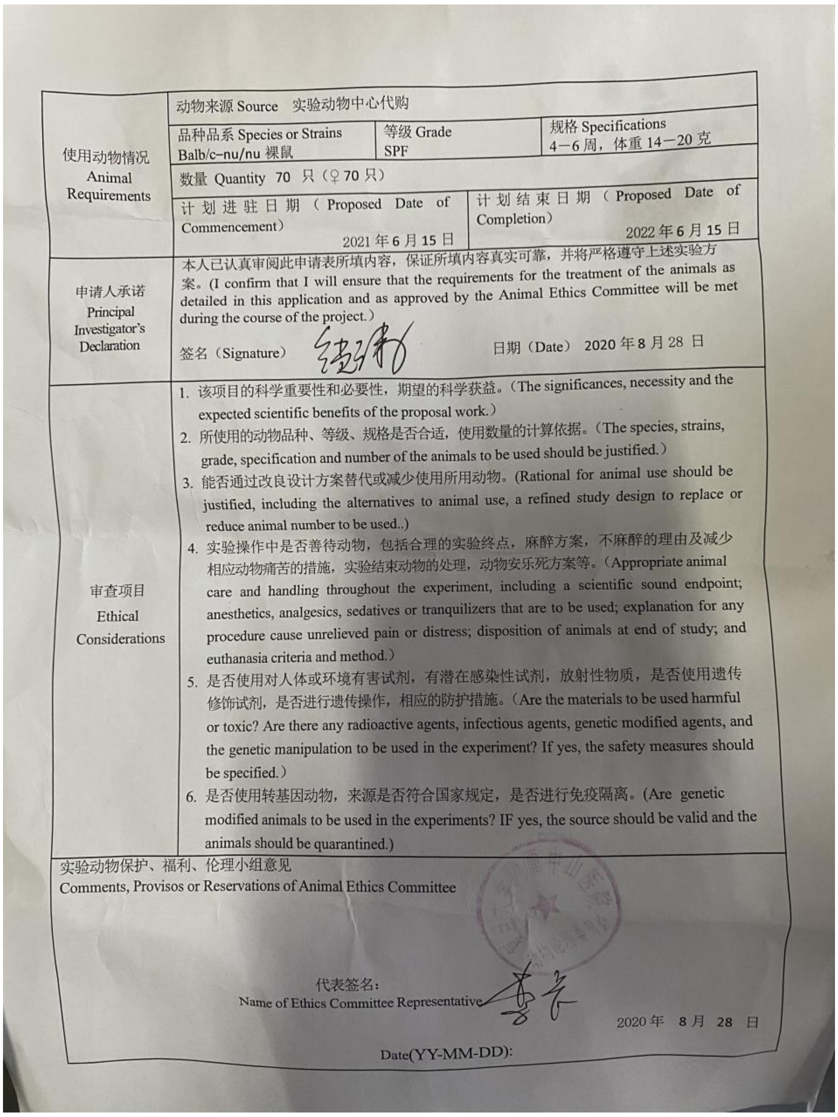

使用动物情况 Animal Requirements	动物来源 Source 实验动物中心代购		
	品种品系 Species or Strains Balb/c-nu/nu 裸鼠	等级 Grade SPF	规格 Specifications 4－6 周，体重 14－20 克
	数量 Quantity 70 只（♀70 只）		
	计划进驻日期（Proposed Date of Commencement） 2021 年 6 月 15 日		计划结束日期（Proposed Date of Completion） 2022 年 6 月 15 日
申请人承诺 Principal Investigator's Declaration	本人已认真审阅此申请表所填内容，保证所填内容真实可靠，并将严格遵守上述实验方案。(I confirm that I will ensure that the requirements for the treatment of the animals as detailed in this application and as approved by the Animal Ethics Committee will be met during the course of the project.)<br>签名（Signature） [signature] 日期（Date） 2020 年 8 月 28 日		
审查项目 Ethical Considerations	1. 该项目的科学重要性和必要性，期望的科学获益。（The significances, necessity and the expected scientific benefits of the proposal work.）<br>2. 所使用的动物品种、等级、规格是否合适，使用数量的计算依据。（The species, strains, grade, specification and number of the animals to be used should be justified.）<br>3. 能否通过改良设计方案替代或减少使用所用动物。(Rational for animal use should be justified, including the alternatives to animal use, a refined study design to replace or reduce animal number to be used..)<br>4. 实验操作中是否善待动物，包括合理的实验终点，麻醉方案，不麻醉的理由及减少相应动物痛苦的措施，实验结束动物的处理，动物安乐死方案等。（Appropriate animal care and handling throughout the experiment, including a scientific sound endpoint; anesthetics, analgesics, sedatives or tranquilizers that are to be used; explanation for any procedure cause unrelieved pain or distress; disposition of animals at end of study; and euthanasia criteria and method.）<br>5. 是否使用对人体或环境有害试剂，有潜在感染性试剂，放射性物质，是否使用遗传修饰试剂，是否进行遗传操作，相应的防护措施。（Are the materials to be used harmful or toxic? Are there any radioactive agents, infectious agents, genetic modified agents, and the genetic manipulation to be used in the experiment? If yes, the safety measures should be specified.）<br>6. 是否使用转基因动物，来源是否符合国家规定，是否进行免疫隔离。(Are genetic modified animals to be used in the experiments? IF yes, the source should be valid and the animals should be quarantined.)		
实验动物保护、福利、伦理小组意见<br>Comments, Provisos or Reservations of Animal Ethics Committee<br>代表签名：<br>Name of Ethics Committee Representative [signature]<br>2020 年 8 月 28 日<br>Date(YY-MM-DD):			

Fig. 1S Animal Ethics Approval
