# Supplementary material for: Fasting-induced RNF152 resensitizes gallbladder cancer cells to gemcitabine by inhibiting mTORC1-mediated glycolysis
Source: iScience. 2024 Apr 8;27(5):109659. doi: 10.1016/j.isci.2024.109659 (PMC11068552; doi:10.1016/j.isci.2024.109659)
Supplement: Table S1. Clinical and demographic information of gallbladder cancer patients [file mmc2.pdf]

Table 1S Clinical and demographic information of gallbladder cancer patients

Case	Gender	Age	Race	LNM	Tumor differentiation	Clinical stage	Pathological diagnosis
**1**	M	47	Asian (China)	Yes	W	3	GBC tissue
**2**	M	60	Asian (China)	Yes	P	3	GBC tissue
**3**	M	66	Asian (China)	Yes	P	3	GBC tissue
**4**	M	39	Asian (China)	Yes	P	3	GBC tissue
**5**	M	73	Asian (China)	No	W	1	GBC tissue
**6**	M	69	Asian (China)	Yes	W	3	GBC tissue
**7**	M	70	Asian (China)	Yes	P	2	GBC tissue
**8**	M	60	Asian (China)	No	W	3	GBC tissue
**9**	M	49	Asian (China)	No	P	2	GBC tissue
**10**	M	55	Asian (China)	Yes	P	3	GBC tissue
**11**	F	47	Asian (China)	Yes	P	3	GBC tissue
**12**	F	54	Asian (China)	Yes	P	3	GBC tissue
**13**	F	73	Asian (China)	No	W	2	GBC tissue
**14**	F	48	Asian (China)	No	W	1	GBC tissue
**15**	F	64	Asian (China)	Yes	P	2	GBC tissue
**16**	F	51	Asian (China)	Yes	P	3	GBC tissue
**17**	F	68	Asian (China)	Yes	P	3	GBC tissue
**18**	F	65	Asian (China)	Yes	P	3	GBC tissue
**19**	F	72	Asian (China)	Yes	W	3	GBC tissue
**20**	F	63	Asian (China)	Yes	W	3	GBC tissue
**21**	F	71	Asian (China)	No	W	1	GBC tissue
**22**	F	52	Asian (China)	Yes	P	3	GBC tissue
**23**	F	51	Asian (China)	Yes	P	2	GBC tissue
**24**	F	46	Asian (China)	Yes	P	3	GBC tissue
**25**	F	69	Asian (China)	No	W	1	GBC tissue

Annotation: M, Male; F, Female; W, Well; P, Poorly; LNM, Lymph node metastasis; GBC, gallbladder cancer.
